# Cellular Patterning of *Arabidopsis* Roots Under Low Phosphate Conditions

**DOI:** 10.3389/fpls.2018.00735

**Published:** 2018-06-05

**Authors:** George Janes, Daniel von Wangenheim, Sophie Cowling, Ian Kerr, Leah Band, Andrew P. French, Anthony Bishopp

**Affiliations:** ^1^Centre for Plant Integrative Biology, School of Biosciences, University of Nottingham, Loughborough, United Kingdom; ^2^Queen's Medical Centre, University of Nottingham Medical School, Nottingham, United Kingdom; ^3^School of Computer Science, University of Nottingham, Nottingham, United Kingdom

**Keywords:** developmental biology, radial patterning, phosphate deficiency, root anatomy, *Arabidopsis*, root hair, cortex, light sheet microscopy

## Abstract

Phosphorus is a crucial macronutrient for plants playing a critical role in many cellular signaling and energy cycling processes. In light of this, phosphorus acquisition efficiency is an important target trait for crop improvement, but it also provides an ecological adaptation for growth of plants in low nutrient environments. Increased root hair density has been shown to improve phosphorus uptake and plant health in a number of species. In several plant families, including Brassicaceae, root hair bearing cells are positioned on the epidermis according to their position in relation to cortex cells, with hair cells positioned in the cleft between two underlying cortex cells. Thus the number of cortex cells determines the number of epidermal cells in the root hair position. Previous research has associated phosphorus-limiting conditions with an increase in the number of cortex cell files in *Arabidopsis thaliana* roots, but they have not investigated the spatial or temporal domains in which these extra divisions occur or explored the consequences this has had on root hair formation. In this study, we use 3D reconstructions of root meristems to demonstrate that the radial anticlinal cell divisions seen under low phosphate are exclusive to the cortex. When grown on media containing replete levels of phosphorous, *A. thaliana* plants almost invariably show eight cortex cells; however when grown in phosphate limited conditions, seedlings develop up to 16 cortex cells (with 10–14 being the most typical). This results in a significant increase in the number of epidermal cells at hair forming positions. These radial anticlinal divisions occur within the initial cells and can be seen within 24 h of transfer of plants to low phosphorous conditions. We show that these changes in the underlying cortical cells feed into epidermal patterning by altering the regular spacing of root hairs.

## Introduction

Phosphorus (P) is a critical macronutrient and essential for plant growth. Unlike nitrogen or ammonium, which are soluble and diffuse through the soil, phosphate (Pi) is immobile within the soil as it often binds clay particles and forms insoluble precipitates (Brady and Weil, 2008). This means that the region around roots becomes quickly depleted of phosphate. Plants have developed several strategies for increasing phosphate acquisition, including changes to root architecture (López-Bucio et al., 2002) and through root hairs (Bates and Lynch, 1996; Ma et al., 2003). Studies in both *Arabidopsis* (using the *root hair defective - rhd2*- mutant) and barley (using the *bald root barley - brb*- mutant) have demonstrated reduced growth in low Pi soils of hairless mutants (Bates and Lynch, 2001; Gahoonia and Nielsen, 2004).

Plants may exhibit different root hair adaptations to low P. For example, tomato, spinach, rape and *Arabidopsis* all exhibit an increase in root hair length under low Pi (Foehse and Jungk, 1983; Ma et al., 2001; Bhosale et al., 2018). However, these species also respond by increasing their root hair density in low Pi conditions. A recent study of root hair traits in 166 accessions of *Arabidopsis thaliana* showed that root hair density and length were not correlated, and that the genotypes that showed the greatest increase in root hair density under low Pi were mostly those that had fewer and shorter root hairs under Pi replete conditions (Stetter et al., 2015).

**Figure 1:**
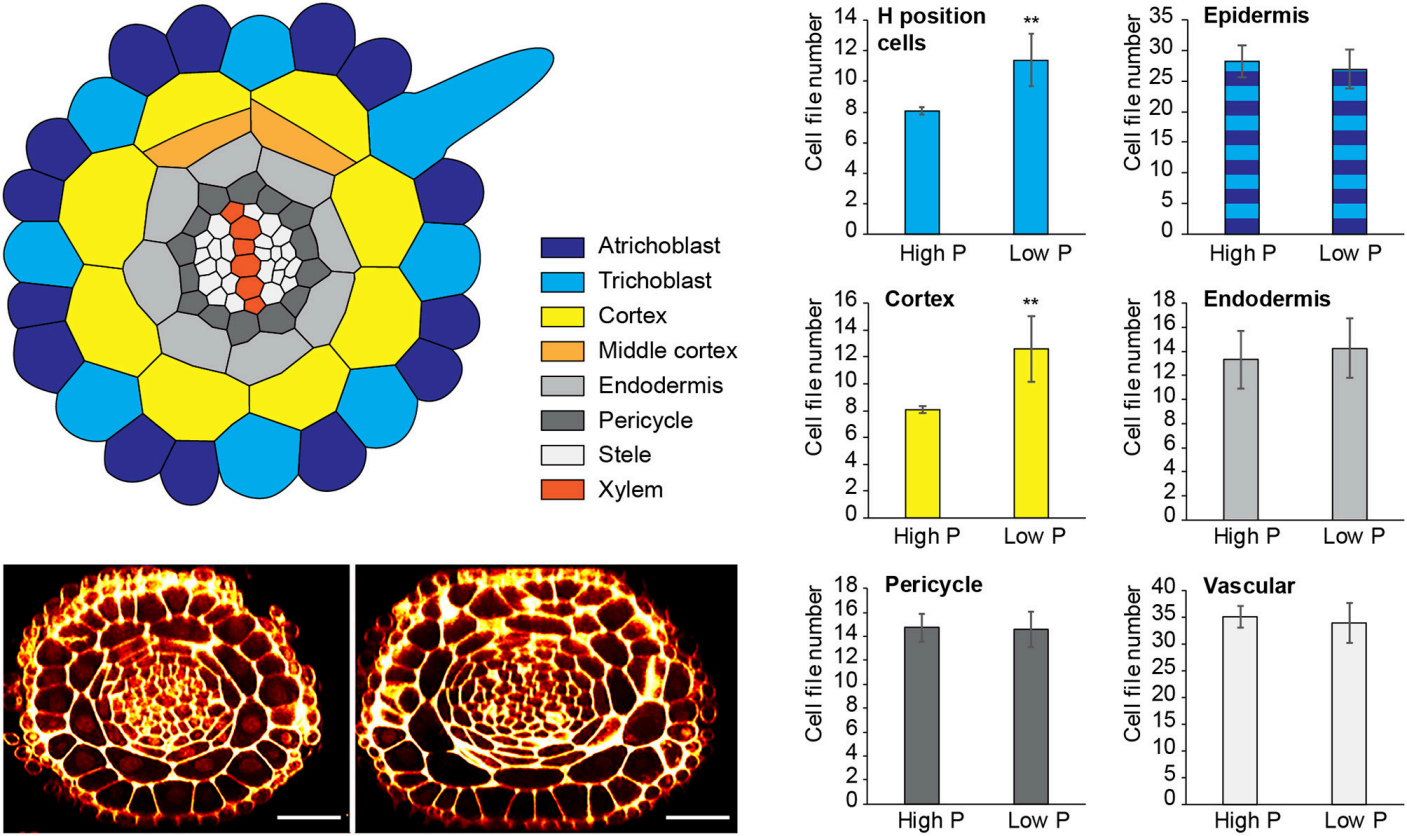
Changes to radial anatomy under low phosphate are limited to changes in cortex and hair-cell number. Schematic diagram showing the radial organization of tissues in the *Arabidopsis* root. Concentric rings of tissues can be seen radiating out from the central vascular cylinder; trichoblast positions in epidermal cells at the cleft between two cortex cells can be seen (light blue). Layers of middle cortex can be seen beginning to form at the xylem poles (orange). Representative images of high Pi **(Left)** and low Pi **(Right)** treated roots in transverse section show how the radial organization differs between treatments, scale bar = 50 μm, Bar charts showing number of cells files in each tissue visualized in confocal microscope cross sections for two phosphate treatments: “high P” (1 mM), “low Pi” (50 μM). Roots of seedlings growing on high Pi (1 mM), or low Pi (50 μM) have, on average, 8 and 12 cortex cell files respectively (*p* ≤ 0.001). This change corresponds to a change in the average number of hair position epidermal cell files (H cells) of 8 to 11 (**p ≤ 0.001).

*Arabidopsis* epidermal cells can acquire one of two fates, they can form trichoblasts that go on to produce root hairs, or they can form atrichoblasts that cannot form root hairs. In wild-type plants, these two cell types form continuous files extending through the root meristem. The cell fate decision is controlled through positional information transmitted from the cortex; trichoblasts form in epidermal cells that overly the cleft between to cortex cells, whilst atrichoblasts overlay just one cortical cell (Figure 1). In wild-type seedlings this results in a radial pattern in which files of trichoblasts are separated by one to three files of non-hair-bearing atrichoblasts (Dolan et al., 1993). The number of cells within each file is different, as trichoblasts are shorter than atrichoblasts (Dolan et al., 1993). Experimental analysis of master regulators of epidermal cell fate has shown that the differences in longitudinal cell length of trichoblasts is dependent upon the position of cells relative to the underlying cortex (Savage et al., 2013).

Pemberton et al. (2001) describe 3 types of epidermal patterning in angiosperms. They refer to the pattern in *Arabidopsis* of hair cells occurring in files separated by one to three files of non-hair cells as type 3. This type of patterning was found in all members of the Brassicaceae examined, as well as other families within the Brassicales and Caryophyllales. Most angiosperms displayed type 1 epidermal patterning, in which all epidermal cells can produce root hairs.

The molecular mechanism regulating epidermal cell fate is well understood in *Arabidopsis*. A transcriptional complex involving the transcription factors - WEREWOLF (WER), GLABRA 2 (GL2), ENHANCER OF GLABRA3 (EGL3), and TRANSPARENT TESTA GLABRA1 (TTGI) is required to specify atrichoblast identity (Grebe, 2012), and mutations in these genes result in plants with increased root hairs (Galway et al., 1994; Rerie et al., 1994; Masucci et al., 1996; Lee and Schiefelbein, 1999; Walker et al., 1999; Bernhardt, 2003). Whereas the transcription factors CAPRICE (CPC), TRIPTYCHON (TRY), and ENHANCER OF CAPRICE (ETC) promote trichoblast identity, and mutations in these genes result in few to no root hairs (Wada et al., 1997; Schellmann et al., 2002; Kirik et al., 2004). Together these components form a regulatory circuit, with CPC moving to trichoblasts where it competes with WER to bind the GL3-EGL3-TTG1 complex (Bernhardt, 2003). JACKDAW (JKD) acts in a non-cell-autonomous manner from the underlying cortex cells to specify trichoblast vs. atrichoblast fate by regulating *GL2, CPC*, and *WER* expression via the LRR kinase SCRAMBLED (SCM) (Kwak and Schiefelbein, 2008; Hassan et al., 2010).

A plant such as *Arabidopsis* that exhibits type 3 root hair patterning could increase root hair density either in the longitudinal domain by a reduction in cell elongation, or in the radial domain by increasing the number of epidermal cells that differentiate as trichoblasts. Indeed, phosphate deficiency has been shown to result in shorter epidermal cells and greater root hair production in *Arabidopsis* (Sánchez-Calderón et al., 2005).

Under normal conditions, *Arabidopsis* roots almost always have exactly eight cortical cells. However, when grown on low Pi media, *Arabidopsis* plants show an increase in the number of cortical cells and this results in an increase in the number of cells at the trichoblast position (Ma et al., 2003; Zhang et al., 2003; Cederholm and Benfey, 2015). Mathematical simulations of epidermal cell fate in which different signal inputs are supplied from the cortex, support a model where a time-evolving signal specifies the recruitment of atrichoblasts from a default trichoblast pathway to control cell length and therefore delay atrichoblast cell fate specification (Savage et al., 2013). These simulations suggest that even under Pi deficient conditions, the core mechanisms regulating epidermal cell fate is functional although plants show increased cell length and form additional trichoblast files (Savage et al., 2013).

In this manuscript, we use a combination of different microscopy techniques to investigate anatomical changes in root radial patterning of *Arabidopsis* in response to low Pi. We show that increases in cortical cell file number occur dynamically within the meristem and respond rapidly to reductions in Pi. Furthermore, we show that these changes in the cortical tissues result in alterations in the final root hair patterning.

## Materials and methods

### Plant material and growth conditions

All *Arabidopsis* experiments were performed using *A. thaliana* (Columbia ecotype) seeds. All seeds were sterilized with 5% sodium hypochlorite for 5 mins before being rinsed with 70% ethanol with 0.01% Triton X-100 once and several times with sterile water. Seeds were then sown on agar plates, sealed with micropore tape (3 M) and cold treated overnight to synchronize germination. Plants were grown under 12 h light, 12 h dark conditions at 21°C. To ensure consistency of our experiments in relation to the photoperiod, all plants were moved into the growth room within the first 2 h of the light period. Harvesting of roots for microscopy and transfer between different media was always done within the same 2 h period at the start of the light cycle.

Low and high P/low and high Fe media was made using a modified Hoaglands medium consisting of the concentrations of minerals listed in Table 1. 0.5g/L of 2-(*N*-morpholino)ethanosulfonic acid (MES) was added and the pH of the media was adjusted to 5.7 using potassium hydroxide. Media was then added to bottles containing Sigma purified agar (catalog number A1296) 8 g/L and autoclaved before being used to pour plates.

**Table 1:** Table showing concentrations of different nutrient components used in low Pi high Fe, high Pi high Fe, low Pi low Fe, and high Pi low Fe media preparations used for low and high P/low and high Fe treatments.

**Nutrient**	**Low Pi high Fe**	**High Pi high Fe**	**Low Pi low Fe**	**High Pi low Fe**
KNO_3_	3 mM	3 mM	3 mM	3 mM
Ca(NO_3_)2 4H_2_O	2 mM	2 mM	2 mM	2 mM
MgSO_4_ 7H_2_O	0.5 mM	0.5 mM	0.5 mM	0.5 mM
(NH_4_)_2_S0_4_	425 μM	0	425 μM	0
NH_4_H_2_PO_4_	50 μM	1 mM	50 μM	1 mM
H_3_BO_3_	25 μM	25 μM	25 μM	25 μM
ZnNa EDTA	2 μM	2 μM	2 μM	2 μM
(NH_4_)_6_Mo_7_O_24_ 4H_2_O	1 μM	1 μM	1 μM	1 μM
KCl	50 μM	50 μM	50 μM	50 μM
MnSO_4_ H_2_O	2 μM	2 μM	2 μM	2 μM
CuSO_4_ 5H_2_O	1 μM	1 μM	1 μM	1 μM
Fe-EDTA	50 μM	50 μM	5 μM	5 μM

### Pseudo-schiff propidium iodide staining

Roots for PS-PI staining were harvested from the plate and fixed immediately in a 50% methanol 10% acetic acid aqueous solution and kept at 4°C overnight. Roots were then rinsed in sterile, deionised water three times before being incubated at room temperature in 1% periodic acid for up to 40 min. Tissue was rinsed 2–3 times with sterile, deionised water and then left in PS PI solution for 1–2 h [0.015 N hydrochloric acid (HCl), 100 mM sodium metabisulphite; 50 μl/ml propidium iodide solution [1 mg/ml], freshly added each time]. Roots were again rinsed 2–3 times with water before being mounted on slides with 40% glycerol 30% chloral hydrate in water and left in the dark over night to clear.

### Microscopy and image processing

Stained cleared roots were imaged using a Leica SP5 confocal microscope. A 488 nm laser was used to excite PI fluorescence for imaging, PI fluorescence was collected using either a photomultiplier tube (PMT) or hybrid detector at between 600 and 800 nm.

For the generation of optical sections from PS-PI stained/cleared roots, Z-stacks of roots were generated using LAS-X Leica software then dissected in orthogonal view using ImageJ image analysis software. An example z stack is shown in Supplemental Movie 1. Cell counts were performed using a cell counter function in the same software. We use post-mitotic cell positions to infer previous cell divisions.

Light-sheet microscopy was performed using a Zeiss Lightsheet Z.1. A 514 nm laser was used to excite YFP as well as propidium iodide. Emission wavelengths were filtered using a bandpass filter BP 525–545 for YFP and BP 575–615 for propidium iodide. Root tips were excised from plants and stained in 5–10 μl propidium iodide (PI)/ml in deionised H_2_O and mounted in to a glass capillary (1 mm inner diameter) using low melting temperature agarose (Sigma-Aldrich) mixed with fluorescent beads from the PS-Speck Microscope Point Source Kit (ThermoFisher Scientific). Roots were imaged along six angles using the Multiview function in ZEN software. The ImageJ plugin Multiview-Reconstruction was used to assemble multiview stacks in to a final stack using fluorescent beads as points of interest for registration (Preibisch et al., 2010, 2014).

Cell files of different cell types were counted by producing radial cross sections of roots from confocal Z-stacks assembled in ImageJ software, viewed in orthogonal views and counted using the cell counter plugin.

## Results

### Changes in radial cell proliferation in response to phosphate are limited to cortex cells

Although previous reports have shown that cortical cell proliferation is increased when *A. thaliana* plants are grown on low phosphate media (Ma et al., 2003; Cederholm and Benfey, 2015), it has been unclear whether the cortex is the only cell type to undergo additional radial anticlinal divisions to increase cell file number. In order to provide a greater understanding of precisely which cell lineages responded to low phosphate, we investigated this in higher resolution by generating 3D reconstructions of the root meristem based on a combination of fixation and labeling with pseudo-Schiff propidium idodide and generating z stacks using confocal microscopy (see Supplemental Movie 1 for an example). *Arabidopsis thaliana* seeds were sown on media containing 1 mM (high P), 50 μM (low P), or 1 μM (very low P) and grown for 12 days. We generated orthogonal projections through roots with which we could count cell file numbers of different cell types (stele, pericycle, cortex, and epidermis) within the meristem (Figure 1). At this age, *Arabidopsis* plants begin to mature, and we see the formation of some middle cortex cells. Previous studies have reported that this is the result of periclinal cell divisions within the endodermal layer (Baum et al., 2002; Lee et al., 2016). We have not included the middle cortex within these cell counts and only count cortex cell files that are in contact with the overlying epidermis.

We found no significant change in the number of stele, pericycle or endodermal cell files or in the total number of epidermal cell files (Figure 1), with the stele having between 30 and 35 cell files, and the pericycle, endodermis, and total epidermal cell files having 14–16, 12–14, and 26–28 cell files respectively. The only tissue that exhibited a change in cell file number was the cortex, where the number of cell files increased from 8 (1 mM P) to an average of 12 (50 μM P) or 11 (1 μM P).

These results show that the radial cell proliferation was restricted to the cortex, and differ from those shown previously by Cederholm and Benfey (2015), who also observed differences in the number of endodermal as well as cortical cells. This could be due to differences in the age of plants analyzed. as this study used 12 day old plants, whereas that by Cederholm and Benfey (2015) used approximately 6 day old plants or may be due to differences in the composition of the media.

### The root cortex response to low phosphate is concentration dependent

Different studies by a variety of groups investigating low Pi stress in *Arabidopsis* seedlings have used a variety of Pi concentrations to mimic a stress scenario. We therefore considered it important to determine the specific concentration of Pi that will trigger cortical cell proliferation, to facilitate comparisons with the plethora of data already existing for architectural and anatomical changes.

**Figure 2:**
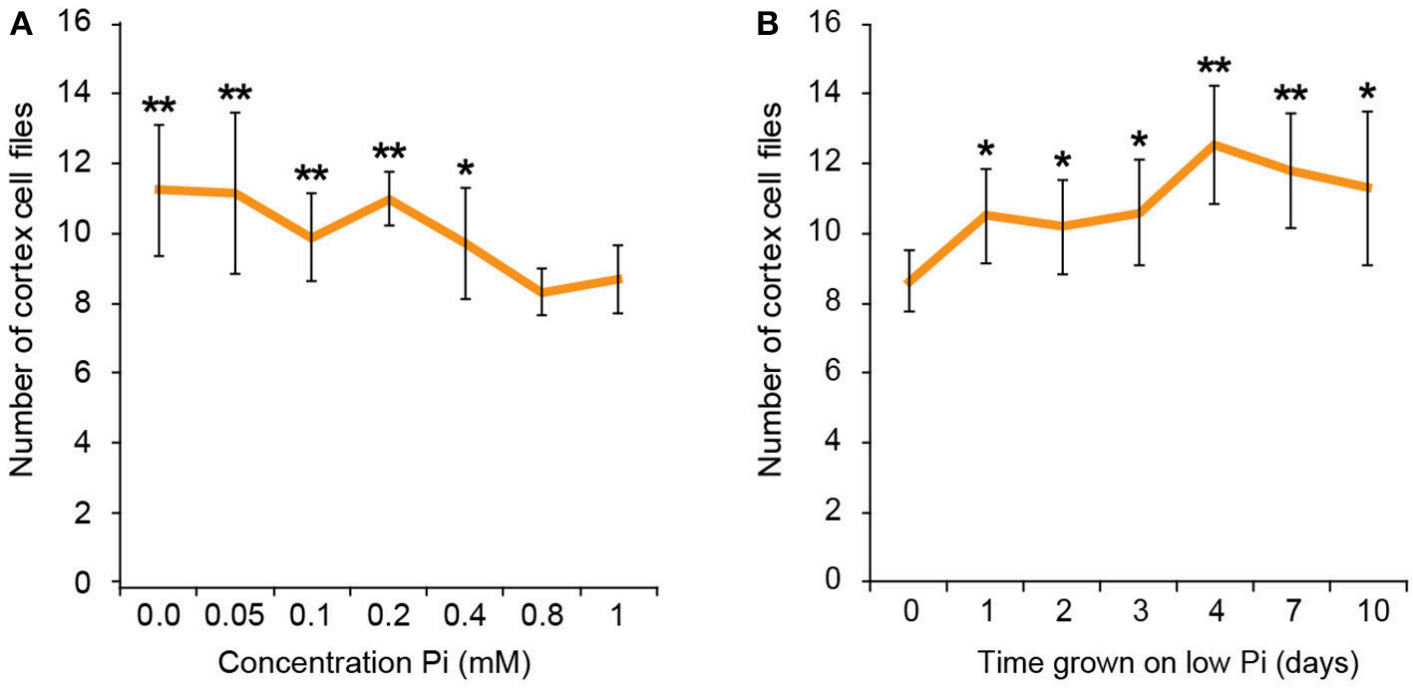
Response to low phosphate is concentration and time dependent. Cortex cell file numbers increase when roots are exposed to medium containing 400 μM or lower. At lower concentrations the number of cortex cell files is as high as 13, but at 800 μM−1 mM the number of cortex cell files remains at 8 **(A)**. Error bars represent standard deviation. Number of plants used in cortex cell file counts 0 M: *n* = 8; 50 μM: *n* = 12; 100 μM: *n* = 11; 200 μM: *n* = 10; 400 μM: *n* = 12; 800 μM: *n* = 11; 1 mM: *n* = 11. ^**^Statistically significant difference to 1 mM Pi (*p* ≤ 0.005); ^*^p ≤ 0.05 **(A)**. Cortex cell file number increases to an average of 10 after 24 h exposure to low Pi, increasing to an average of 12 after 4 days. **(B)** Error bars represent standard error. Numbers of plants used in cell file counts 0 days: *n* = 11; 1 day: *n* = 10; 2 days: *n* = 11; 3 days: *n* = 10; 4 days: *n* = 9; 7 days: *n* = 10; 10 days: *n* = 10. ^**^Statistically significant difference to 1 mM Pi (*p* ≤ 0.005); *p ≤ 0.05.

To find the lowest concentration of phosphate, at which a response in cortical cell file number (CCFN) was seen, plants were grown under a concentration range from 0 M to 1 mM Pi. All concentrations between 0 and 400 μM Pi elicited an increase in CCFN. Within this range there was little difference in the number of cell files, with 0 M, 50, 100, 200, and 400 μM all showing ~10–11 cortex cell files (Figure 2). Root length and lateral root density was also measured for these plants, and it was observed that plants grown on <50 μM phosphate typically exhibited more severe stress symptoms than those grown on higher concentrations, including dramatic inhibition of growth, anthocyanin accumulation and chlorosis. After 9 days root lengths were 69 ± 9 mm on high Pi and 26 ± 1 mm on low Pi. These general plant phenotypes were similar to those previously reported for plants grown in phosphate deficient conditions, such as Bates and Lynch (1996) and Trull et al. (1997). These results show that the changes in CCFN occur at Pi concentrations that do not severely affect plant growth, suggesting that this is an adaptive response rather than simply a disorganization of the meristem in response to phosphate starvation.

It has been shown previously that iron concentration in media affects root architectural responses to low phosphate (Hirsch et al., 2006; Svistoonoff et al., 2007; Ward et al., 2008; Dong et al., 2017; Gutiérrez-Alanís et al., 2017) as under low Pi conditions roots can accumulate high concentrations of free iron in the root meristem. This accumulation seems to be particularly high in the QC and surrounding initials and has been suggested to have a toxic effect that can lead to meristem disorganization and mis-localisation of key patterning regulators such as the transcription factors SHR and SCR (Ticconi et al., 2004, 2009). Whilst there is increasing evidence that iron toxicity may be responsible for some of the extreme responses observed for *Arabidopsis* plants grown on agar plates under phosphate limiting conditions (such as a severe reduction in primary root length). More recent studies have mitigated this iron toxicity effect when using media with <1 μM Pi by reducing iron levels concomitantly with Pi reduction.

**Figure 3:**
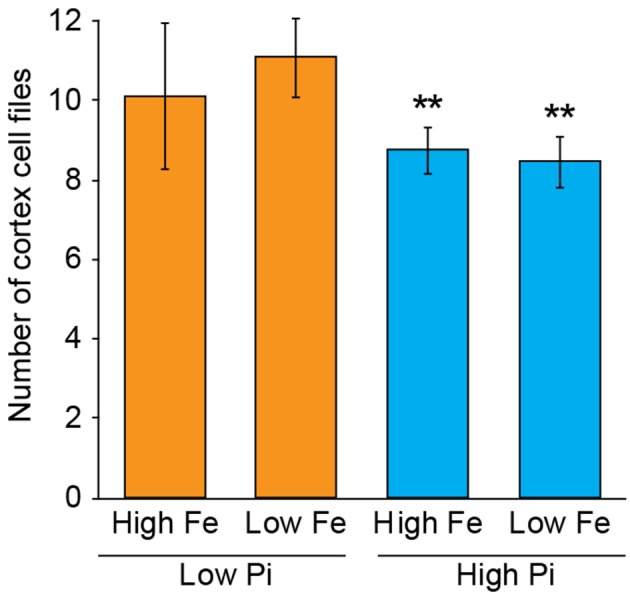
Exposure to low Pi with low iron maintains the CCFN response of *Arabidopsis* roots to low Pi. Pseudo-Schiff propidium iodide stained roots imaged using confocal microscopy to generate optical transverse cross sections. Bar chart showing average CCFN for each treatment: LPHFe, CCFN = 10, *n* = 11; LPLFe, CCFN = 11, *n* = 11; HPHFe, CCFN = 8.75, *n* = 12; HPLFe, CCFN = 8.45, *n* = 11. Error bars represent standard deviation. ^**^Statistically significant difference from HPHFe (*p* ≤ 0.001); ^*^statistically significant difference from HPHFe (*p* ≤ 0.05).

To test whether the response in CCFN to reduced phosphate in the media was primarily associated with a lack of Pi or an excess of iron, we set up experiments with *A. thaliana* sown on to agar plates containing media with high Pi high Fe (1 mM NH_4_H_2_PO_4_, 50 μM Fe-EDTA), high Pi low Fe (1 mM NH_4_H_2_PO_4_, 5 μM Fe-EDTA), low Pi high Fe (50 μM NH_4_H_2_PO_4_, 50 μM Fe-EDTA), and low Pi low Fe (50 μM NH_4_H_2_PO_4_, 5 μM Fe-EDTA). We counted CCFN in all these lines, and observed that when plants are exposed to low Pi, but also 10-fold less iron (5 μM Fe-EDTA) an increase in CCFN was still present. On this media, roots have a CCFN of 11; a result that is higher than on low Pi high Fe (Figure 3). This data supports the hypothesis that alterations in CCFN seen in Pi limiting conditions are primarily a response to low Pi and not due to an increase in Fe and/or Fe toxicity in the root.

### Changes to radial cortex anatomy occur after 24 H exposure to low phosphate just above the cortex endodermal initial

**Figure 4:**
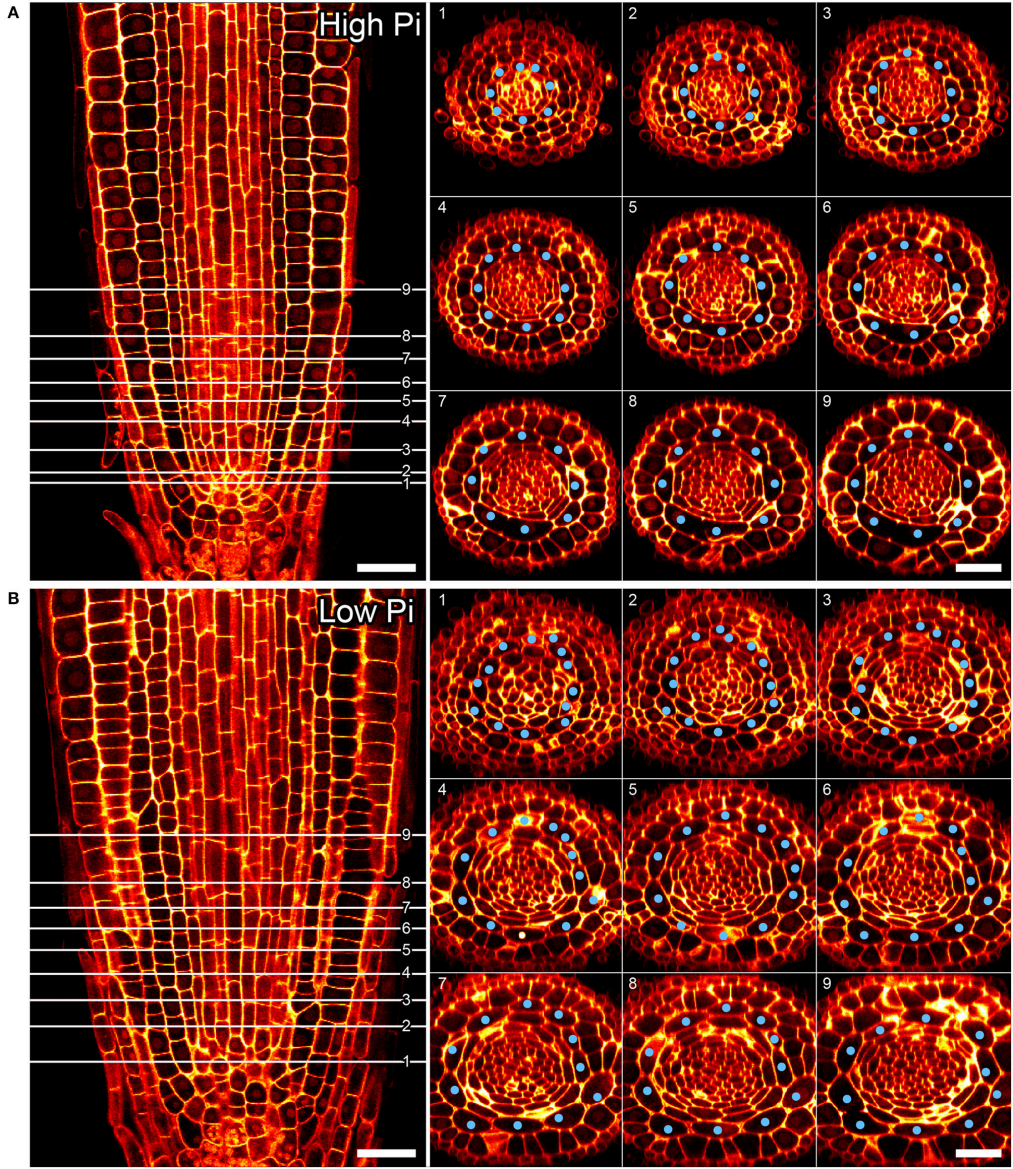
Cortical cell proliferation occurs close to the root apical meristem. Reconstructed confocal Z-stacks of imaged pseudo-Schiff propidium iodide stained roots show that cortex cell file (blue spots) numbers are highest in proximity to the growing tip/cortex endodermis initial (CEI) cells in roots grown on 50 μM phosphate **(B)**. Plant roots grown on 1 mM phosphate containing medium exhibit 8 cortex cell files from 1 to 10 cell tiers (white lines) behind the cortex endodermal initial cell **(A)**. Cell file counts high P: *n* = 17; low Pi *n* = 30, differences between low and high Pi statistically significant *p* ≤ 0.001. Scale bar = 25 μm.

**Figure 5:**
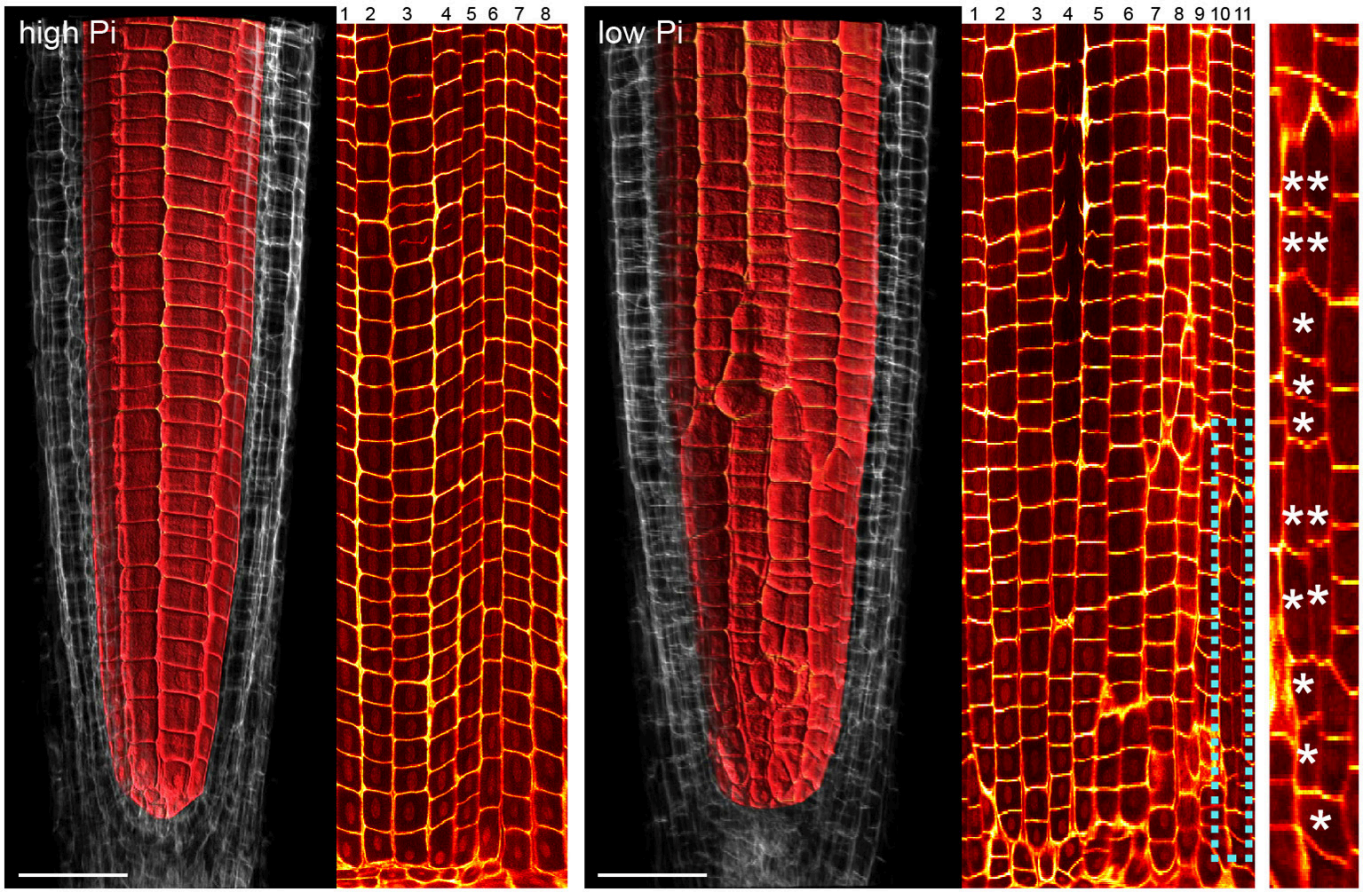
Cell lineage analyses reveal multiple cell division events leading to increases in radial anticlinal cortical cell file number. Unrolled images showing cell lineages in the cortex of *Arabidopsis* roots under high Pi **(Left)** and low Pi **(Right)**. Using Adobe After Effects we digitally dissected the membranes between cortex cells. In ImageJ. We unrolled this data set in order to visualize the cortical cell lineages within the root meristem. The dissected cell layers unrolled are shown in Supplemental Movie 2. Whilst 8 continuous cell files can be traced from just above the quiescent center until the start of the elongation zone for plants grown on high Pi, when grown on low Pi, we observe multiple events in which CCFN is increased in relatively small numbers of cells. These events are marked with asterisks. Scale bars = 50 μm.

Previous studies have shown that cortex proliferation in response to low Pi in *Arabidopsis* roots is visible within the root tip. However none of this work provided an explanation as to where exactly in the root meristem the proliferative divisions take place. In order to address this gap in the knowledge, we assembled 3-dimensional image stacks using confocal microscopy on chloral hydrate cleared pseudo-Schiff propidium iodide stained root tips grown on low or high P. By assembling these image stacks in ImageJ we determined the number of cortex cell files at specific cell tiers behind the quiescent center. Counting the number of cortex cell files at each tier revealed a trend for decreasing CCFN with distance from the QC in low Pi treated roots (Figure 4). Under high Pi conditions, CCFN was consistently 8 at each of the 10 tiers distal to the QC (Figure 4). However under low Pi the average number of CCFs at tier one is around 12, which then decreases to 11 at tier 10 (Figure 4). In order to reveal a clear view on the cortex cells only, we isolated the cortex cells from the rest of the cell layers by manually drawing a black mask in Adobe After Effects. We unrolled this layer (using ImageJ's function RadialReslice) to produce a longitudinal image of the cortical cell lineages (Figure 5 and Supplemental Movie 2). Whilst 8 continuous cell files can be traced from just above the quiescent center until the start of the elongation zone for plants grown on high Pi, when grown on low Pi, we observe multiple events in which CCFN is increased in relatively small numbers of cells. From these images it was concluded that the proliferative divisions occur within the meristem, and as we saw many independent events altering CCFN in a relatively small longitudinal domain. We speculate that this is likely to be a dynamic processes perhaps responding to local levels of Pi.

We next asked if the changes in CCFN observed on low phosphate were a dynamic event capable of adapting rapidly to a low phosphate environment. We reasoned that if this cortical re-patterning is to confer an adaptive advantage to, then it should occur quickly, for example in the case of a root grows though a patch of soil low in P. To address the dynamics of this response, seedlings were grown on plates containing replete levels of Pi (1 mM). Plants were then transferred to 50 μM Pi containing medium after 4, 7, 10, 11, 12, and 13 days. Twenty four hours after the final transfer, when seedlings were 14 days old, roots were harvested from all the 50 μM Pi plates, alongside control plants from 1 mM Pi plates and roots imaged using pseudo-Schiff staining coupled with confocal microscopy. Strikingly, we observed a CCFN response to low Pi within 1 day of transferring plants to low Pi (Figure 4). Within this timeframe we saw a statistically significant increase in average CCFN from 8.6 to 10.5.

After this time point, the average CCFN remains between 9.5 and 12.5 with a small amount of variation depending on the transfer stage (Figure 3). These findings are broadly in line with other changes in plant response to low Pi as they occur much slower than changes in gene response. For example, changes in the expression of transcription factors such as WRKY75 occur within 3 h of transfer to low phosphate media (Devaiah et al., 2007).

### Proliferation of cortical cells alters epidermal patterning

Previously, it had been hypothesized that altering cortical cell file number would provide a mechanism through which root hair density can be increased in the radial dimension. We examined positions of epidermal cells laying in the H (trichoblast) position (i.e., spanning a junction between two cortical cells) and the N position (i.e., overlaying just one cortical cell). We found that the number of epidermal cells in the H position increased from 8 (1 mM P) to 11 (50 μM P) under Pi limiting conditions (Figures 1, 4B). These results support previous observations by other researchers (Ma et al., 2001).

**Figure 6:**
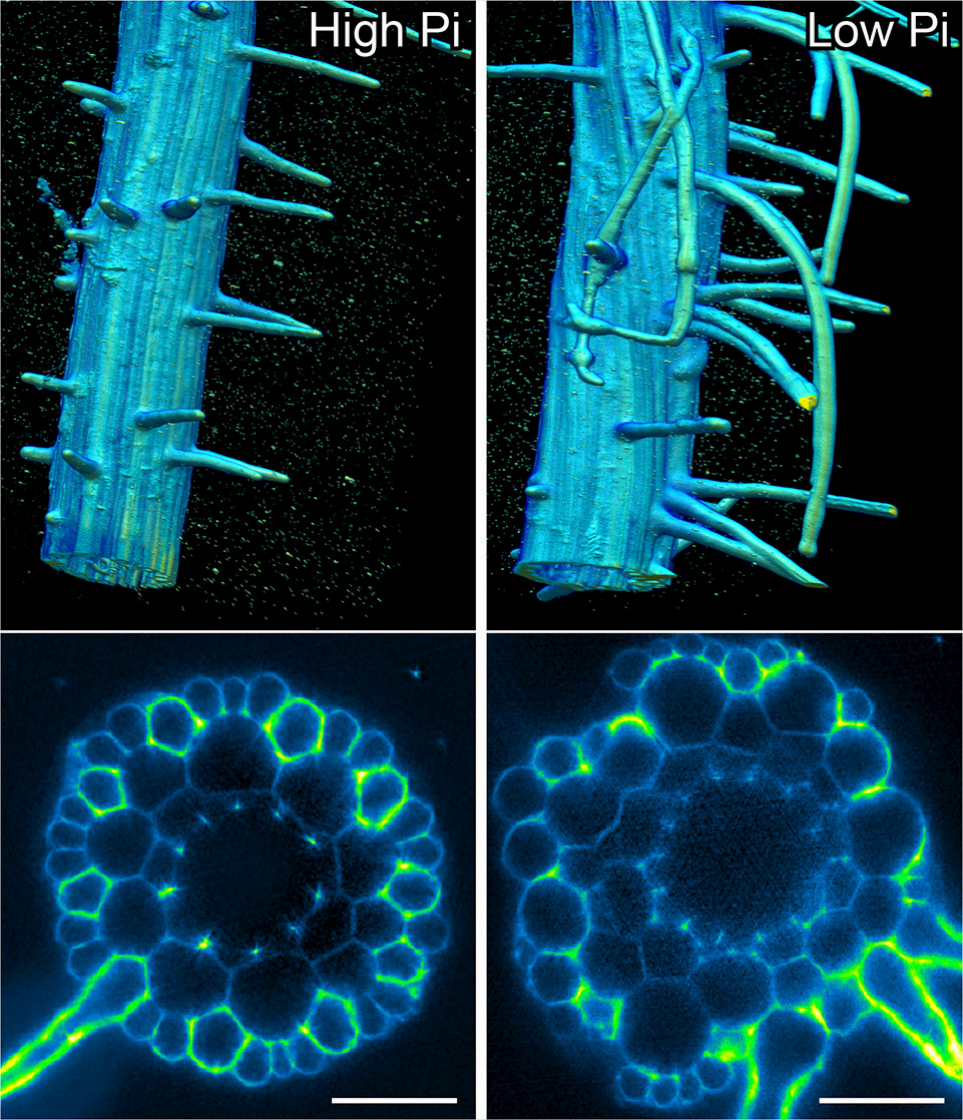
Light sheet imaging reveals atypical root hair patterning scenarios under low Pi. Anatomy of a mature part of *Arabidopsis* under high Pi **(Left)** and low Pi conditions **(Right)**. Multi-view recording along six directions captured using Light Sheet Fluorescence Microscopy. A 3D reconstruction (Arivis Vision 4D software) of the fused image stack is shown in the upper panel and a single slice cross section is presented in the lower panel. Roots were stained with propidium iodide. Scale bars = 50 μm.

**Figure 7:**
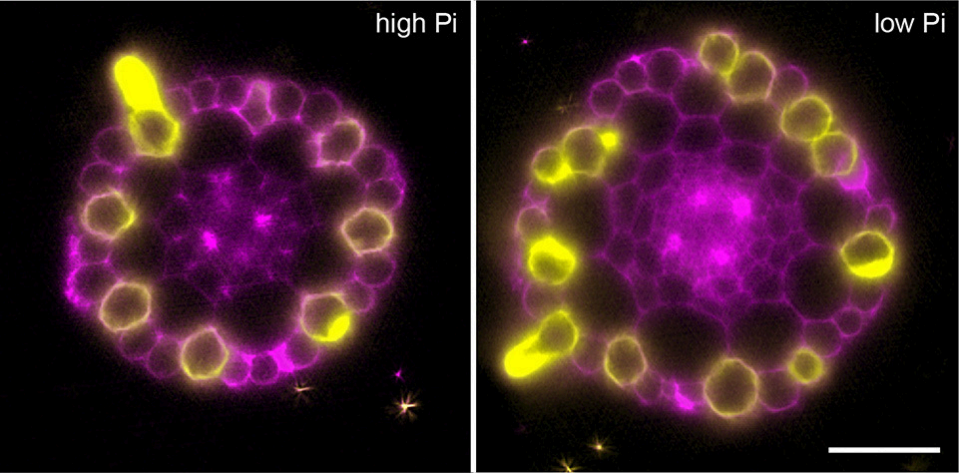
Expression of the COBL9 marker shows atypical specification of trichoblast identity under low Pi. Anatomy of a mature part of *Arabidopsis* under high Pi **(Left)** and low Pi conditions **(Right)**. Multi-view recording along six directions captured using Light Sheet Fluorescence Microscopy to visualize the pCOBL9::GFP gene (yellow) and propidium iodide (magenta). Scale bars = 50 μm.

**Figure 8:**
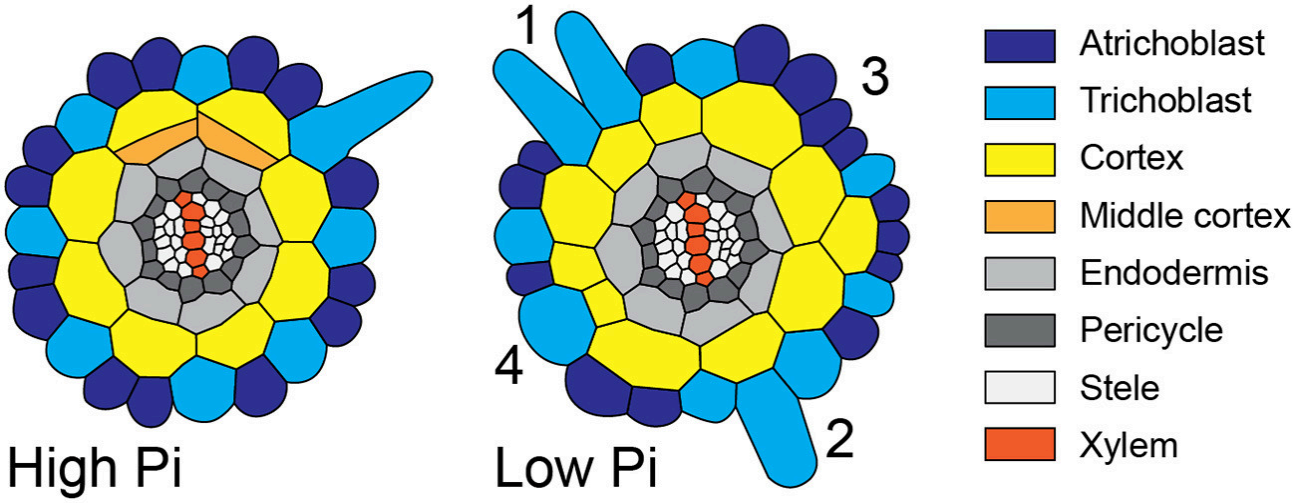
Schematic diagram showing atypical root hair patterning scenarios found under low Pi conditions. Schematic representations of root radial cross sections under high Pi **(Left)** and low Pi **(Right)** conditions showing changes in tissue patterning including four atypical root hair patterning scenarios. These scenarios are: (1) the formation of 2 adjacent hair cells files forming over two adjacent cortical cells clefts, (2) the formation of a hair cell in a non-hair-cell position, (3) no root hairs forming from an H-cell file, and (4) one H cell file forming over two adjacent cortical cell clefts.

In order to confirm that this change in positional information for a selected subset of epidermal cells was sufficient to alter cell fate specification in these cells, we used light sheet microscopy to fully reconstruct 3D representations of mature tissues to evaluate the radial distribution of both expression of the root hair marker COBRA-like *COBL9* (Roudier et al., 2002) and the presence of the root hairs themselves in context of the underlying cortical cell geometry. We used light sheet microscopy because it enables a large volume to be imaged rapidly, it can be used with living samples and it does not require any clearing protocol which could change the cellular structural integrity. When plants were grown on high phosphate we observed a pattern as previously reported with files of trichoblasts over clefts between cortical cells, with these files being separated by 1–3 files of atrichoblasts (Figures 6–8 and Supplemental Movies 3–5). We only occasionally saw deviations from this stereotypical pattern, with for example, occasional ectopic expression of *COBL9* in epidermal cells overlaying a single cortex cell. When plants were grown under low phosphate we saw a breakdown of this regular spacing and frequently found four non-typical root hair patterning scenarios. The first scenario was that we saw adjacent files of trichoblasts as a result of two neighboring epidermal cells overlying two adjacent cortex cell clefts (Figure 5). In reconstructions of roots grown on low Pi, we not only observed root hair forming cells forming at positions overlaying a cleft between two cortical cells, but in all reconstructions, we saw several examples where epidermal cells both expressing *COBL9* and forming root hairs were found over a single cortical cell (Figures 6–8 and Supplemental Movies 3–5), which we denoted as scenario 2. We also observed several instances in which epidermal cells lying in the cleft between two cortex cells neither express *COBL9* nor form root hairs (scenario 3). Finally, the fourth scenario we noted was that of root hairs emerging from a file of epidermal cells overlaying cleft between more than two cortical cells. For roots grown on high or low Pi we observe examples in which cells expressing *COBL9* either fail to form root hairs or only produce very short root hairs, although this is likely to come in part from an asymmetry in the availability of water imposed by the agar plate (Bao et al., 2014). Using a conventional microscopy approach we measured the root hair length on the side of the root facing away from the agar plate and observed an increase in root hair length for plants grown on low Pi (High Pi, 153 ± 73 μm; Low Pi, 270 ± 128 μm) Collectively, these results suggest that rather than simply increasing trichoblast number, alterations in the cortical cell patterning file number have significant effects in which the regular root hair patterning mechanism of the mature root becomes unstable and a more chaotic pattern emerges (Figures 6–8).

## Discussion

*Arabidopsis thaliana* roots have previously been shown to respond to low phosphate by producing extra cortical cells (Ma et al., 2001; Cederholm and Benfey, 2015). However, although this phenomenon had been reported, it had not been explored in great detail. For example, it was unclear if the cortex was the only cell type to show increased cell file number under low Pi, it was unknown which cortical cells underwent extra cell divisions, the exact timing of this response was unknown and it was unclear whether this was a specific response to low Pi or a result of iron toxicity. Furthermore, although changes in CCFN were reported, these were not correlated experimentally with alterations in root hair density or patterning.

To address these issues we exploited two methods for generating 3D reconstructions of the root meristem for plants grown under high and low Pi conditions. The first involved fixing roots and using a modified pseudo Schiff reagent to stain cell walls with propidium iodide. These stained roots were then visualized on a confocal microscope. This technique allowed us to image around 20–30 roots for each sample in a medium throughput manner. This confocal based approach allowed us to build upon the original data produced by Ma et al. (2003) that used just 6 roots. The second approach was based upon analysis using light sheet microscopy of roots stained with propidium iodide. In order to fully resolve the entire volume of the mature root we captured Z-stacks along six different angles coupled with the bead-based multiview reconstruction alignment (Preibisch et al., 2010). Four roots grown on low Pi and four roots grown on high Pi were analyzed, as well as a further 2 roots on high Pi and 3 roots on low Pi expressing the *COBL9* marker. This has allowed us to observe that trichoblasts can form in positions overlaying a single cortical cell, in plants grown on low Pi; a phenomenon that has not previously been described.

Collectively our data builds upon previous work showing files of extra cortical cells throughout the meristem (Cederholm and Benfey, 2015) by demonstrating that several rounds of radial anticlinal divisions occur close to the root apex. Our experiments transferring plants from Pi replete to Pi limiting conditions show that anatomical changes occur quickly, with the first changes occurring within 24 h of transfer. These results are in keeping with those required of an adaptive response evolved to maximize Pi acquisition from soil. For a root to maximize Pi capture from a heterogeneous soil environment, the developmental program controlling root hair patterning must be able to adapt quickly to changes in the local environment at a range of concentrations. We observed that changes in CCFN occurred at Pi concentrations between 0 and 400 μM, with the strongest effect seen at 50 μM. This was surprising as this concentration is far above what is seen is most soils (Raghothama, 1999), but this could be explained as the availability of Pi on plates is likely to be very different from that in the soil. Recently the relevance of traditional gel-based systems for investigating the effects of phosphate limitation on root growth has been challenged, and a new system has been developed in which Pi is adsorbed onto Al_2_0_3_ particles (Hanlon et al., 2018). This has the advantage of delivering buffered levels of Pi to roots at concentrations closer to the Pi levels of natural soils. Although there are changes to *Arabidopsis* root architecture and anatomy when roots are grown on buffered vs. non-buffered medium, increases in CCFN on low Pi medium have been observed with both systems (Hanlon et al., 2018). This supports existing data showing that changes in CCFN are a direct response to low Pi, however it also indicates that alterations in the responses seen at different concentrations of Pi in this study cannot be interpreted literally and do not represent levels found within soils.

Whilst our results were generally in keeping with those published previously (Ma et al., 2003; Cederholm and Benfey, 2015), they differed in a few minor aspects. Cederholm and Benfey (2015) reported changes in the number of endodermal cells. However in our assay the numbers of vascular cells, pericycle cells, endodermal cells and total epidermal cells did not differ significantly between low and high Pi conditions. We also observed changes in cortical cell file number on transfer to Pi limiting conditions earlier than has been reported by other authors. Cederholm and Benfey (2015) report changes in cortical cell file number at 2 days post-transfer, Ticconi et al. (2009) report changes in ground tissue in the Pi hypersensitive phosphate *deficient root 2* (*pdr2*) mutant after 2 days of transfer; whilst we observed the first changes occurring after only 24 h.

One limitation for performing studies limiting phosphate on agar plates, is that under low Pi conditions, roots can accumulate high concentrations of free iron in the root meristem (Hirsch et al., 2006; Svistoonoff et al., 2007; Ward et al., 2008; Dong et al., 2017; Gutiérrez-Alanís et al., 2017). This can have a toxic effect leading to meristem disorganization and ultimately termination. Our data demonstrates that the increased cortical cell file number, is direct a result of low Pi rather than extra iron.

Although increased CCFN has been proposed to offer an advantage to improving Pi acquisition under low nutrient conditions, it is only one of several traits employed by *Arabidopsis* to increase root hair density, as decreased epidermal cell size impacts root hair density in the longitudinal dimension and increased root hair length can increase uptake of Pi from the soil (Foehse and Jungk, 1983; Ma et al., 2001). However, when we followed the radial distribution of root hairs and compared it with the underlying pattern imposed by the cortex, we observed alterations in the regular spacing of root hair cell rather than a general increase. We frequently, observed multiple files of root hairs forming adjacently (scenario 1), we observed root hairs forming in positions where the epidermal cells overlay just one cortical cell (scenario two), epidermal cells overlaying a cleft between two cortical cells producing no root hairs (scenario three), and root hairs forming from single epidermal cell files overlying two adjacent cortical cell clefts (scenario four). These scenarios are summarized schematically in Figure 8. However, we did not see a significant increase in the radial density of root hairs. Our results challenge the assumption that the trait of increased CCFN has evolved in species that exhibit type 3 root hair patterning as a mechanism specifically to increase root hair density, although it is possible that different effects could be observed at different concentrations of Pi.

It is important to develop understanding of the mechanisms through which roots respond to low nutrient environments in order to develop new lines that can utilize nutrients existing in the soil more efficiently. In this paper, we perform a detailed anatomical study of how radial anatomy is altered under low phosphorus in *Arabidopsis* and the effect that modulating cortical cell file number has on controlling density of root hairs in the radial dimension. This study will inform future programmes that may seek to modulate root hair density in plants similar to *Arabidopsis* as it challenges the utility of focusing on CCFN. It opens up interesting questions regarding whether increased cortical cell file number offers an alternative adaptive advantage to growth on low Pi, and provides an intriguing example for future studies about how patterning events in one tissue can affect patterning in others.

## Author contributions

AB, GJ, and DvW designed experiments. GJ, SC, and DvW performed experiments and analyzed results. AB, GJ, and DvW wrote the manuscript with input from LB, AF, and IK.

### Conflict of interest statement

The authors declare that the research was conducted in the absence of any commercial or financial relationships that could be construed as a potential conflict of interest.
